# Novel Technique to Increase the Effective Workspace of a Soft Robot

**DOI:** 10.3390/mi15020197

**Published:** 2024-01-28

**Authors:** Gerardo I. Pérez-Soto, Karla A. Camarillo-Gómez, Juvenal Rodríguez-Reséndiz, Carlos G. Manríquez-Padilla

**Affiliations:** 1 Facultad de Ingeniería, Universidad Autónoma de Querétaro, Santiago de Querétaro 76010, Mexico; israel.perez@uaq.mx (G.I.P.-S.); juvenal@uaq.edu.mx (J.R.-R.); 2 Departament of Mechanical Engineering, Tecnológico Nacional de México en Celaya, Celaya 38010, Mexico; karla.camarillo@itcelaya.edu.mx; 3 Facultad de Ingeniería, Universidad Autónoma de Querétaro, San Juan del Río 76807, Mexico

**Keywords:** soft robotics, tensegrity robot, FEM-ANSYS, form-finding

## Abstract

This article presents a novel technique for a class 2 tensegrity robot, also classified as a soft robot, to increase workspace by increasing the number of geometric equilibrium configurations of the robot. The proposed modification, unlike the strategies reported in the literature, consists of increasing the number of points where the flexible and rigid elements that make up the robot come into contact without the need to increase the number of actuators, the number of flexible elements, or modify the geometry of the rigid elements. The form-finding methodology combines the basic principles of statics with the direct and inverse kinematic position analysis to determine the number of equilibrium positions of the modified robot. In addition, numerical experiments were carried out using the commercial software ANSYS^®^, R18.2 based on the finite element theory, to corroborate the results obtained with them. With the proposed modification, an increase of 23.369% in the number of geometric equilibrium configurations is achieved, which integrates the workspace of the modified class 2 tensegrity robot. The novel technique applied to tensegrity robots and the tools developed to increase their workspace apply perfectly to scale the robots presented in this paper.

## 1. Introduction

The term tensegrity was used for the first time by Buckminster Fuller et al. in 1975 [1]; it comes from the combination of the words “tensional” and “integrity”, referring to structures that maintain their shape when subjected to internal tension and compression loads by all the elements that comprise them. One of the most accepted definitions for tensegrity systems is the one proposed by A. Pugh [2], where he defines them as a set of discontinuous elements subject to compression loads that interact with a set of continuous elements subject to tension loads, forming a stable volume in space; the discontinuous elements are called bars, and the continuous ones are denominated flexible elements. Incorporating various types of actuators, such as McKibben artificial muscles [3], which enable the variation of the length of flexible elements into a tensegrity structure, creates tensegrity robots. The actuators incorporated into tensegrity robots can be powered by various sources of motion, such as EHD pumps [4], piezoelectric materials [5], and cable–pulley systems [6], among others. Several robots designed based on the concept of tensegrity systems have been reported in the literature. A notable example is a robot with a fundamental tensegrity structure known as the Snelson Cross, proposed by J. Begay et al. [7]. Additionally, robots inspired by more intricate tensegrity structures have been documented. These include an arm-type tensegrity robot with multiple elementary tensegrity structures connected in series [8,9]. Worm-like mobile robots with multiple movement modes and minimal actuators have also been proposed, as demonstrated by Y. Jin et al. [10]. Furthermore, there are reports of mobile robots equipped with wheels inspired by the dynamics of the human spine for duct inspection, such as the one developed by F. Carreño and M.A. Post [11]. Also, a platform-type robot based on a structure with variable stiffness and deployable capabilities has been developed by D. Zappetti et al. [12], among others.

**Figure 1:**
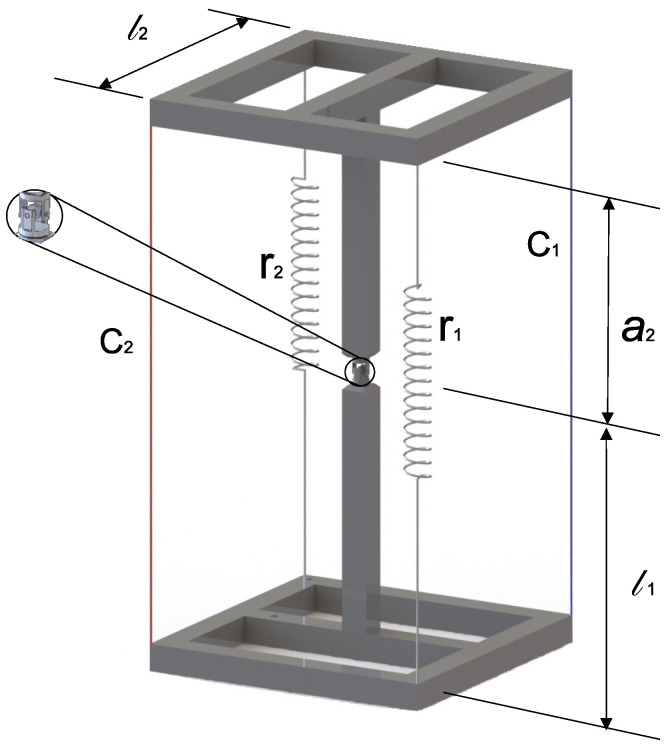
Class 2 tensegrity robot.

C.G. Manríquez-Padilla et al. [13] proposed a new robot based on a tensegrity structure, which, according to the classification proposed by R. E. Skelton et al. [14], is classified as a tensegrity class 2 structure. The proposed robot comprises two rigid substructures joined at one of their ends by a universal joint and, at the remaining ends, using four flexible elements, marked as $C_{1}$, $C_{2}$, $r_{1}$, and $r_{2}$, as shown in Figure 1.

In Figure 1, the flexible elements marked as $r_{1}$ and $r_{2}$ represent tension springs, while the flexible elements marked as $C_{1}$ and $C_{2}$ represent cables. The cable lengths are variable and are controlled by turning a pulley attached to a servomotor.

A form-finding analysis must be performed to determine the workspace of the robot, that is, the set of possible geometric configurations that the robot can reach or between which one can migrate without collapsing. Several strategies have been reported in the literature that aim to solve the form-finding analysis; among them, there are some based on neural networks [15], the Levenberg–Marquardt method [16], methods based on force density matrix [17], some others based on optimization algorithms [18], and genetic algorithms combined with potential energy minimization [19]. For the class 2 tensegrity robot reported in [13] and illustrated in Figure 1, C.G. Manríquez-Padilla et al. [20] proposed a solution strategy for the form-finding analysis based on a combination of the fundamental principles of statics and the direct and inverse kinematic position analysis using the geometric parameters proposed by Denavit–Hartenberg [21]. In the same study, a case was presented wherein 14,641 geometric configurations were analyzed, and only 215 were classified as equilibrium geometric configurations.

Tensegrity-structures-based robots, by combining soft materials with rigid materials, present considerable challenges when designing and building them, especially when it is necessary to modify their workspace. As outlined in the previously mentioned literature, the modification of the workspace for tensegrity structures composed of elementary tensegrity structures (tetrahedra, dodecahedra, icosahedra, etc.) is accomplished by connecting several elementary structures at their ends, thereby increasing the number of elements that compose the robot. The methodology mentioned above cannot be applied to tensegrity robots with more complex structures that are not composed of elementary tensegrity structures because the base structure lacks a homogeneous distribution of elements. Consequently, the strategies to modify the workspace of intricate tensegrity-structures-based robots have not been fully defined due to the complexity of the interaction of the elements [22]. Therefore, based on the outcomes presented in [20], this paper introduces a redesign of the class 2 tensegrity robot. The redesign involves altering the mode of interaction between the flexible and rigid elements comprising the class 2 tensegrity robot, as depicted in Figure 1. The novelty of this proposed redesign aims to augment the number of equilibrium geometric configurations and, thereby, enhance the workspace of the robot, all without requiring an increase in the number of servomotors or flexible elements.

To achieve this goal, in Section 2.1, a brief description of the modified class 2 tensegrity robot is presented, followed by the description of the Denavit–Hartenberg parameters used to develop the kinematic position analysis. Section 2.4 introduces the development of the kinematic position analysis for the modified robot and the methodology corresponding to the inverse kinematic position analysis. Subsequently, the static analysis and the form-finding analysis are presented. Section 3 and Section 4 present the numerical experiments of a study case using commercial software based on finite element theory, ANSYS^®^R18.2. The results from the numerical experiments are used to corroborate the proposed redesign of the class 2 tensegrity robot.

## 2. Methodology

**Figure 2:**
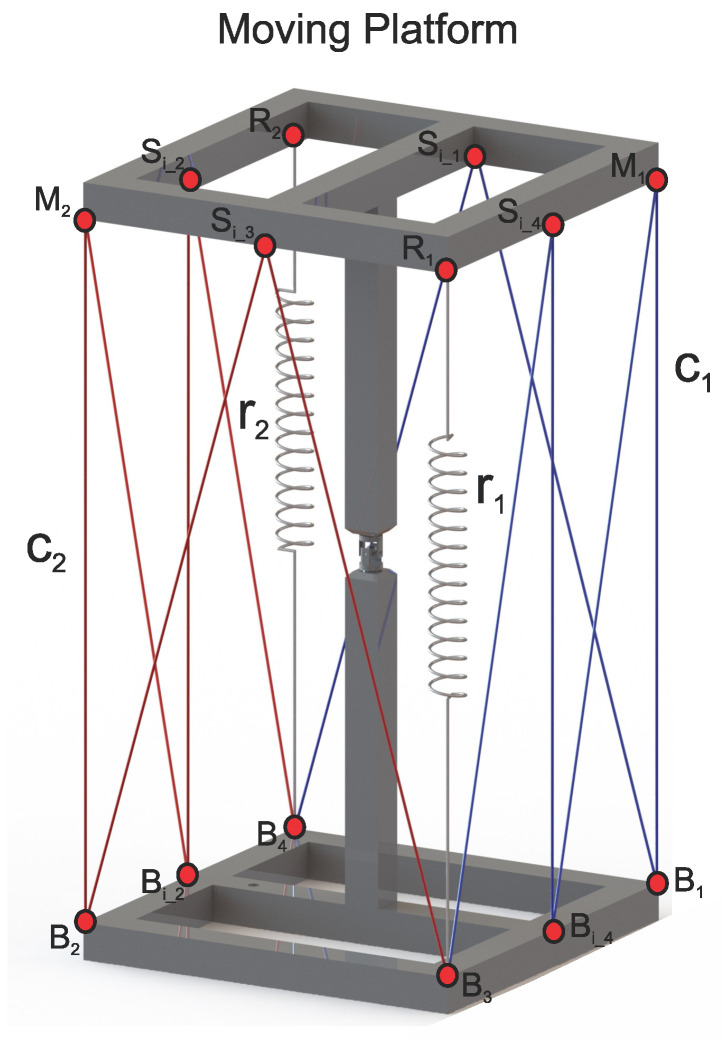
Proposed class 2 tensegrity robot.

This section presents a detailed description of the novel technique proposed to increase the workspace of a class *k* tensegrity robot. To achieve this, the modification proposed for the class 2 tensegrity robot shown in Figure 2 and the technique developed to solve the form–finding problem are detailed. Unlike the strategies to increase the workspace reported in the literature, the proposed technique does not require increasing the number of actuators, modifying the kinematic structure of the robot, or increasing the number of elastic elements that compose it.

### 2.1. Description of the Modified Class 2 Tensegrity Robot

The following argument is presented as a hypothesis to increase the number of equilibrium geometric configurations for a class 2 tensegrity robot: “*The increase in the number of points where the flexible elements come into contact with both the fixed base and the mobile platform is proportional to the increase in the number of equilibrium geometric configurations*”.

To increase the contact points between the flexible and rigid elements of the class 2 tensegrity robot, it is necessary to attach a pulley system that allows the cables with variable lengths, $C_{1}$ and $C_{2}$, shown in Figure 1, to be in contact with the moving platform and the fixed base at multiple points. The proposed design of this pulley system is shown in Figure 2.

Figure 2 depicts the flexible elements highlighted in blue and red, corresponding to cables with variable lengths $C_{1}$ and $C_{2}$, respectively. Furthermore, the flexible elements labeled as $r_{1}$ and $r_{2}$ represent tensional springs with constant stiffness. Points $M_{1}$ and $M_{2}$, marked on the mobile platform, define the original interaction points between the rigid and flexible elements found in both robots; the robot is shown in Figure 1, and the proposed robot is shown in Figure 2. In the same way, points labeled $S_{i\_1}$, $S_{i\_2}$, $S_{i\_3}$, and $S_{i\_4}$ represent the points of interaction between the rigid and flexible elements that were added to increase the workspace. Similarly, on the fixed base, points labeled $B_{1}$, $B_{2}$, $B_{3}$, $B_{4}$, $B_{i\_2}$, and $B_{i\_4}$, represent all the points where the cables with variable lengths $C_{1}$ and $C_{2}$ come into contact with the fixed base. The pulley systems are located on the points described above. For the present work, the pulley system is considered a friction-free system, designed to allow the free interaction between the flexible elements, cables with variable lengths $C_{1}$ and $C_{2}$, and the rigid elements of the mobile platform and the fixed base.

### 2.2. Kinematic Position Analysis of the Modified Class 2 Tensegrity Robot

**Figure 3:**
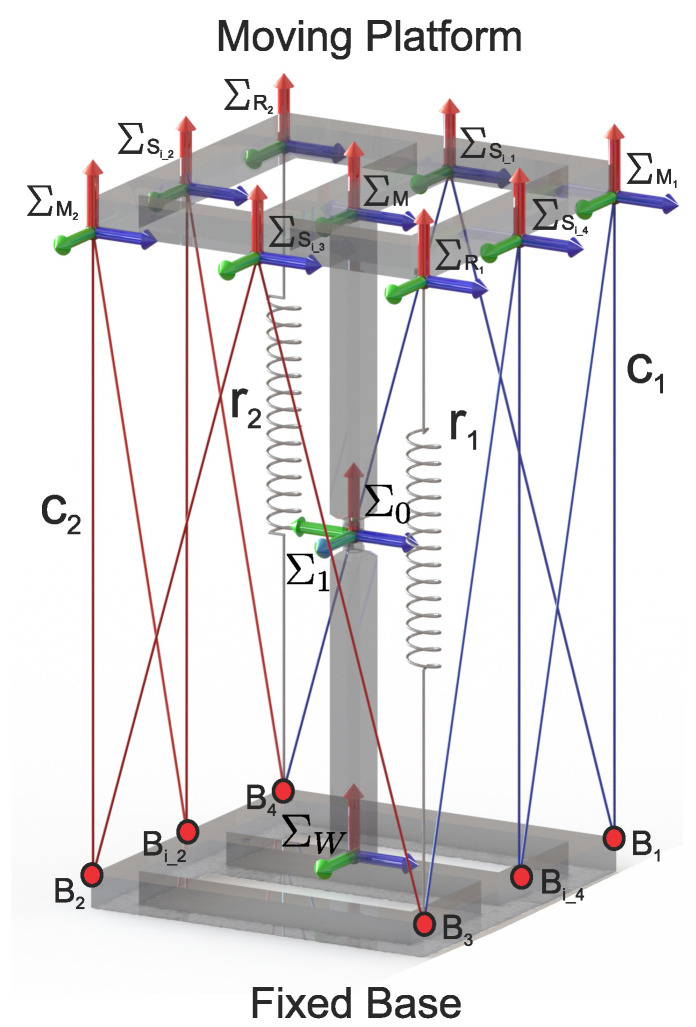
Coordinate reference systems attached to the proposed robot.

**Figure 4:**
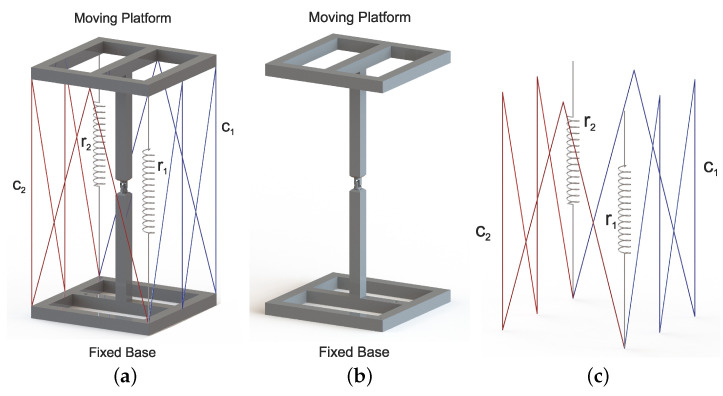
Decouple class 2 tensegrity robot. (**a**) Modified robot. (**b**) Rigid part. (**c**) Flexible part.

For the proposed class 2 tensegrity robot, shown in Figure 3, the methodology described in [13] was implemented, which consists of performing a kinematic decoupling of the robot to carry out the direct and inverse kinematic position analysis for both the flexible and rigid elements independently. Decoupling the tensegrity robot is carried out in two parts: the rigid elements shown in Figure 4b and the flexible elements shown in Figure 4c; the joint variables of the universal joint can be related with the variation in the lengths of the flexible elements.

#### 2.2.1. Denavit–Hartenberg Parameters

For the modified class 2 tensegrity robot, the Denavit–Hartenberg distal parameters [23] establish the reference frames shown in Figure 3, coloring the axes *x*, *y*, and *z* as red, green, and blue, respectively.

Table 1 shows the Denavit–Hartenberg distal parameters of the proposed class 2 tensegrity robot, where

- $d_{i}$ is the distance measured from the $x_{i-1}$ axis to the $x_{i}$ axis along the $z_{i-1}$ axis.
- $\theta_{i}$ is the angle between the $x_{i-1}$ axis and the $x_{i}$ axis measured around the $z_{i-1}$ axis, following the right-hand convention.
- $a_{i}$ is the distance measured from the $z_{i-1}$ axis to the $z_{i}$ axis along the $x_{i}$ axis.
- $\alpha_{i}$ is the angle between the $z_{i-1}$ axis and the $z_{i}$ axis measured around the $x_{i}$ axis, following the right-hand convention.

**Table 1:** Denavit–Hartenberg parameters for the robot.

*i*	$d_{i}$	$\theta_{i}$	$a_{i}$	$\alpha_{i}$
	mm	rad	mm	rad
1	0	$\theta_{1}$	0	−π/2
2	0	$\theta_{2}$	$a_{2}$	π/2

For the kinematic position analysis, it is necessary to define ten auxiliary reference frames, as shown in Figure 3, where the reference frame $\Sigma_{W}$ is called the reference frame of the fixed base, and the reference frame $\Sigma_{M}$ is considered the terminal organ of the modified class 2 tensegrity robot. The remaining reference frames are used to describe the position of the points where the flexible elements are in contact with the mobile platform of the robot with respect to $\Sigma_{W}$. To accomplish this, the origin of the reference frames, $\Sigma_{M_{1}}$, $\Sigma_{M_{2}}$, $\Sigma_{S_{i\_1}}$, $\Sigma_{S_{i\_2}}$, $\Sigma_{S_{i\_3}}$, $\Sigma_{S_{i\_4}}$, $\Sigma_{r_{1}}$, and $\Sigma_{r_{2}}$, must be in the same position as the points $M_{1}$, $M_{2}$, $S_{i\_1}$, $S_{i\_2}$, $S_{i\_3}$, $S_{i\_4}$, $r_{1}$, and $r_{2}$, respectively, as shown in Figure 2.

By design criteria, the coordinates of the points $B_{1}$, $B_{2}$, $B_{3}$, $B_{4}$, $B_{i\_2}$, and $B_{i\_4}$ are known with respect to $\Sigma_{W}$. Considering the displacement of the fixed base as non-existent, it can be said that the coordinates of all points located on it are known and invariant in time.

#### 2.2.2. Direct Kinematic Position Analysis

The kinematic analysis of the proposed class 2 tensegrity robot, shown in Figure 4a, serves to describe the position and orientation of the mobile platform of the tensegrity robot with respect to the $\Sigma_{W}$ reference frame as a function of the variable joints that show the variations in the lengths of cables $C_{1}$ and $C_{2}$. Considering the above, it is necessary to define the variations in the cable lengths, $C_{1}$ and $C_{2}$, as a function of the degrees of rotation of joints 1 and 2 described by the variables $\theta_{1}$ and $\theta_{2}$.

To analyze the rigid elements, the distal Denavit–Hartenberg parameters contained in Table 1 are used in combination with the homogeneous transformation matrix for the distal variant [23], described by

(1) Tii−1=Cθi−CαiSθiSαiSθiaiCθiSθiCαiCθi−SαiCθiaiSθi0SαiCαidi0001

where $C\theta_{i}\equiv \cos\left(\theta_{i}\right)$, $S\theta_{i}\equiv \sin\left(\theta_{i}\right)$, $C\alpha_{i}\equiv \cos\left(\alpha_{i}\right)$, $S\alpha_{i}\equiv \sin\left(\alpha_{i}\right)$. Substituting the values of Table 1, in the Equation (1), the homogeneous transformation matrices T10 and TM1 are obtained and defined by

(2) T10=Cθ10−Sθ10Sθ10Cθ100−1000001

(3) TM1=Cθ20Sθ2a2Cθ2Sθ20−Cθ2a2Cθ201000001

In addition, the auxiliary reference frames described in Figure 3 are used to obtain the homogeneous transformation matrices that define the position and orientation of the points located on the mobile platform shown in Figure 2 using

(4) T0W=100l1010000100001

(5) TM2M=1000010−l2*001l2*0001

(6) TR2M=1000010−l2*001−l2*0001

(7) TSi_2M=1000010−l2*00100001

(8) TSi_4M=1000010l2*00100001

(9) TM1M=1000010l2*001−l2*0001

(10) TR1M=1000010l2*001l2*0001

(11) TSi_1M=10000100001−l2*0001

(12) TSi_3M=10000100001l2*0001

where T0W∈R4×4 describes the homogeneous transformation matrix that relates the reference frame $\Sigma_{0}$ with respect to $\Sigma_{W}$; conversely, the homogeneous transformation matrices TM1M,TM2M,TR1M,TR2M, TSi_1M,TSi_2M,TSi_3M, TSi_4M∈R4×4 relate the auxiliary reference frames $\Sigma_{M_{1}}$, $\Sigma_{M_{2}}$, $\Sigma_{r_{1}}$, $\Sigma_{r_{2}}$, $\Sigma_{S_{i\_1}}$, $\Sigma_{S_{i\_2}}$, $\Sigma_{S_{i\_3}}$, $\Sigma_{S_{i\_4}}$ with the reference frame $\Sigma_{M}$. The variables $l_{1}$ and $l_{2}$ represent the distance between the axis $z_{W}$ and $z_{0}$ measured along the axis $x_{W}$ and the length of the mobile platform measured along the axis *y* as shown in Figure 1, respectively. Also, $l_{2}^{*}=\frac{l_{2}}{2}$ is defined.

By multiplying (2)–(4), the homogeneous transformation matrix TMW is obtained, and it describes the position and orientation of the reference frame $\Sigma_{M}$ with respect to $\Sigma_{W}$, i.e., [24],

(13) TMW=T0WT10TM1=Cθ1Cθ2−Sθ1Cθ1Sθ2l1+a2Cθ1Cθ2Sθ1Cθ2Cθ1Sθ1Sθ2a2Cθ2Sθ1−Sθ20Cθ2−a2Sθ20001

Similarly, by multiplying (9)–(13), the position and orientation of the reference frames located on the mobile platform of the robot are obtained, Figure 3, relative to $\Sigma_{W}$, i.e.,

(14) TM1W=TMWTM1M

(15) TM2W=TMWTM2M

(16) TR1W=TMWTR1M

(17) TR2W=TMWTR2M

(18) TSi_1W=TMWTSi_1M

(19) TSi_2W=TMWTSi_2M

(20) TSi_3W=TMWTSi_3M

(21) TSi_4W=TMWTSi_4M

**Figure 5:**
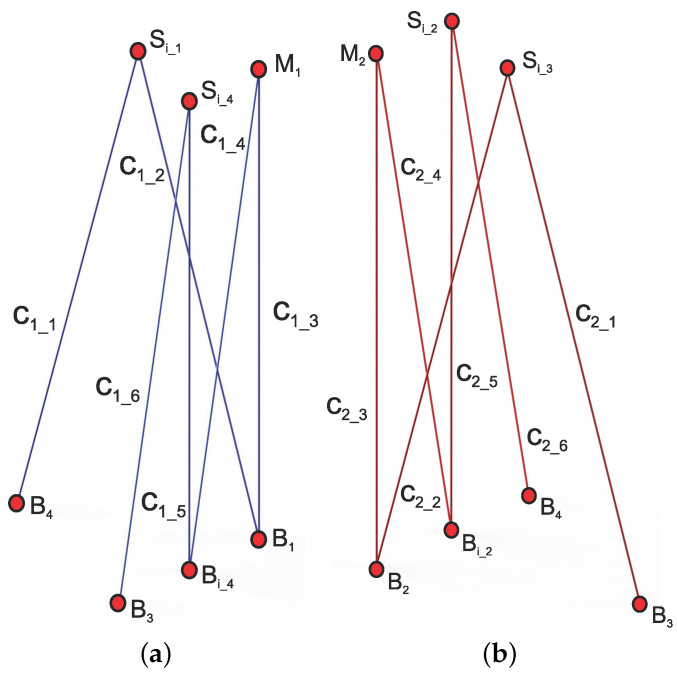
Segmentation of cables $C_{1}$ and $C_{2}$: (**a**) cable segments $C_{1}$; (**b**) cable segments $C_{2}$.

Considering the above, as shown in Figure 5, the total lengths of cables $C_{1}$ and $C_{2}$ are defined as

(22) $$\begin{array}{ccc} L_{C_{1}} & = & \sum_{i=1}^{6}L_{C_{1\_i}} \end{array}$$

(23) $$\begin{array}{ccc} L_{C_{2}} & = & \sum_{i=1}^{6}L_{C_{2\_i}} \end{array}$$

where $L_{C_{1\_i}}$ and $L_{C_{2\_i}}$ represent the individual lengths of each of the six segments that make up cables $C_{1}$ and $C_{2}$, respectively. To determine the individual lengths of the cable segments $C_{i\_j}$, the coordinates of the contact points $B_{1}$, $B_{2}$, $B_{3}$, $B_{4}$, $B_{i\_2}$, $B_{i\_4}$, with respect to $\Sigma_{W}$, are assumed to be known based on the dimensions of the tensegrity robot, provided by

(24) PB1W=B1xB1yB1zT

(25) PB2W=B2xB2yB2zT

(26) PB3W=B3xB3yB3zT

(27) PB4W=B4xB4yB4zT

(28) PBi_2W=Bi_2xBi_2yBi_2zT

(29) PBi_4W=Bi_4xBi_4yBi_4zT

Then, the position vectors of the homogeneous transformation matrices (14)–(21) with respect to the fixed base reference frame $\Sigma_{W}$ are provided by

(30) PMjW=PMjxWPMjyWPMjzWT

(31) PSi_kW=PSi_kxWPSi_kyWPSi_kzWT

where j=1,2 and k=1,2,…,4. Using the position vectors described in (24)–(29), the position vectors describing the cable segments are obtained as

(32) C1_1=C1_1xC1_1yC1_1z=(PSi_1xW−B4x)(PSi_1yW−B4y)(PSi_1zW−B4z)

(33) C1_2=C1_2xC1_2yC1_2z=(PSi_1xW−B1x)(PSi_1yW−B1y)(PSi_1zW−B1z)

(34) C1_3=C1_3xC1_3yC1_3z=(PM1xW−B1x)(PM1yW−B1y)(PM1zW−B1z)

(35) C1_4=C1_4xC1_4yC1_4z=(PM1xW−Bi_4x)(PM1yW−Bi_4y)(PM1zW−Bi_4z)

(36) C1_5=C1_5xC1_5yC1_5z=(PSi_4xW−Bi_4x)(PSi_4yW−Bi_4y)(PSi_4zW−Bi_4z)

(37) C1_6=C1_6xC1_6yC1_6z=(PSi_4xW−B3x)(PSi_4yW−B3y)(PSi_4zW−B3z)

(38) C2_1=C2_1xC2_1yC2_1z=(PSi_3xW−B3x)(PSi_3yW−B3y)(PSi_3zW−B3z)

(39) C2_2=C2_2xC2_2yC2_2z=(PSi_3xW−B2x)(PSi_3yW−B2y)(PSi_3zW−B2z)

(40) C2_3=C2_3xC2_3yC2_3z=(PM2xW−B2x)(PM2yW−B2y)(PM2zW−B2z)

(41) C2_4=C2_4xC2_4yC2_4z=(PM2xW−Bi_2x)(PM2yW−Bi_2y)(PM2zW−Bi_2z)

(42) C2_5=C2_5xC2_5yC2_5z=(PSi_2xW−Bi_2x)(PSi_2yW−Bi_2y)(PSi_2zW−Bi_2z)

(43) C2_6=C2_6xC2_6yC2_6z=(PSi_2xW−B4x)(PSi_2yW−B4y)(PSi_2zW−B4z)

Using (32)–(43), the length of the individual segments of each cable as a function of the joint variables, $\theta_{1}$ and $\theta_{2}$, is defined as

(44) $$\begin{array}{c} \begin{array}{c} L_{C_{i\_j}}=\sqrt{{\left(C_{{i\_j}_{x}}\right)}^{2}+{\left(C_{{i\_j}_{y}}\right)}^{2}+{\left(C_{{i\_j}_{z}}\right)}^{2}} \end{array} \end{array}$$

Finally, using (44) for i=1,2 and j=1,2,3,…,6 and substituting in (22) and (23), the total length of the wires $C_{1}$ and $C_{2}$ are obtained based on the variables $\theta_{1}$ and $\theta_{2}$.

#### 2.2.3. Inverse Kinematic Position Analysis

The objective of the inverse kinematic position analysis is to be able to describe the variations in the lengths of cables $C_{1}$ and $C_{2}$ given a desired configuration of the tensegrity robot. Therefore, the above is possible once the solutions for the variables $\theta_{1}$ and $\theta_{2}$ are obtained, corresponding to the desired robot configuration.

Considering that the position and orientation of the reference frame $\Sigma_{M}$, shown in Figure 3, is defined by the known homogeneous transformation matrix:

(45) TMW=t11t12t13xmt21t22t23ymt31t32t33zm0001

where (45) is related to the symbolic matrix provided in (13), the variables $\theta_{1}$ and $\theta_{2}$ can be obtained by

(46) $$\begin{array}{ccc} \theta_{1} & = & \mathrm{atan}2\left(\frac{t_{23}}{t_{13}}\right) \end{array}$$

(47) $$\begin{array}{ccc} \theta_{2} & = & \mathrm{atan}2\left(\frac{-t_{31}}{t_{33}}\right) \end{array}$$

Then, variables $\theta_{1}$ and $\theta_{2}$, obtained through (46) and (47), respectively, are substituted into (44) and evaluated for i=1,2 and j=1,2,3,…,6. Finally, (22) and (23) are substituted into (44) to determine the lengths of the individual cable segments, providing the total lengths of cables $C_{1}$ and $C_{2}$, respectively.

### 2.3. Static Analysis for the Class 2 Tensegrity Robot

Static analysis is the set of operations to calculate the magnitude of the internal forces, ${\vec{f}}_{i}$, that act on the *i*–elements that make up the tensegrity robot, whose input data are the values of the variables, $\theta_{1}$ and $\theta_{2}$. Static analysis is particularly useful to determine if a specific geometric configuration can be considered as an equilibrium geometric configuration, that is, a geometric configuration where the center of mass of the robot does not present movement.

**Figure 6:**
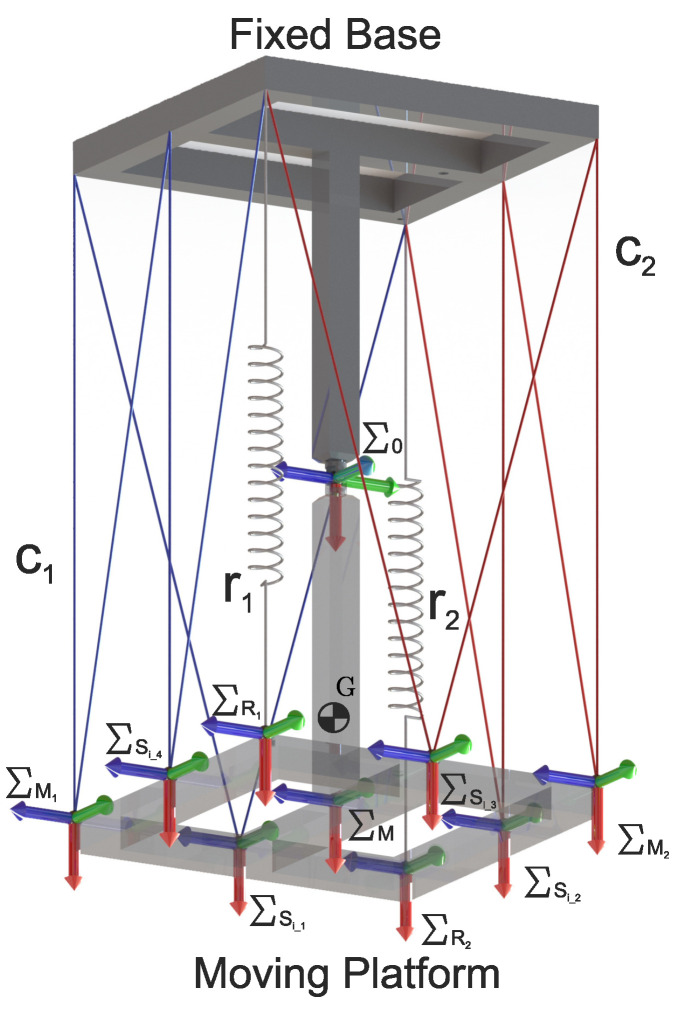
Reference frames used in the static analysis of the modified class 2 tensegrity robot.

For analysis purposes, consider the geometric configuration of the class 2 tensegrity robot shown in Figure 6.

It is considered that the weight of the mobile platform is concentrated in its centroid, G. As a reference point for the calculation of displacements, forces, and moments, frame $\Sigma_{0}$ is used; furthermore, the position and orientation of the reference frame $\Sigma_{M}$ is used to describe the desired geometric configuration.

Notice that the following data are known:

- Total lengths, $L_{C_{1}}$ and $L_{C_{2}}$, corresponding to cables $C_{1}$ and $C_{2}$, respectively.
- The lengths, $L_{r_{1}}$ and $L_{r_{2}}$, corresponding to the springs $r_{1}$ and $r_{2}$, respectively.
- The position and orientation of the reference frames, $\Sigma_{M}$, $\Sigma_{M_{1}}$, $\Sigma_{M_{2}}$, $\Sigma_{S_{i\_1}}$, $\Sigma_{S_{i\_2}}$, $\Sigma_{S_{i\_3}}$, $\Sigma_{S_{i\_4}}$, $\Sigma_{R_{1}}$, and $\Sigma_{R_{2}}$, with respect to the $\Sigma_{W}$ reference frame.

Furthermore, as design parameters are known:

- The lengths of the undeformed springs, $L_{r_{1_{0}}}$ and $L_{r_{2_{0}}}$, corresponding to the tension springs $r_{1}$ and $r_{2}$, respectively.
- The position of the centroid, G, corresponding to the moving platform with respect to the reference frame $\Sigma_{W}$.
- The total mass of the robot.

Then, through the static analysis of the tensegrity robot, the values of the stiffness constants that ensure an equilibrium position, $k_{1}$, and $k_{2}$, can be established, corresponding to the springs $r_{1}$ and $r_{2}$, respectively.

**Figure 7:**
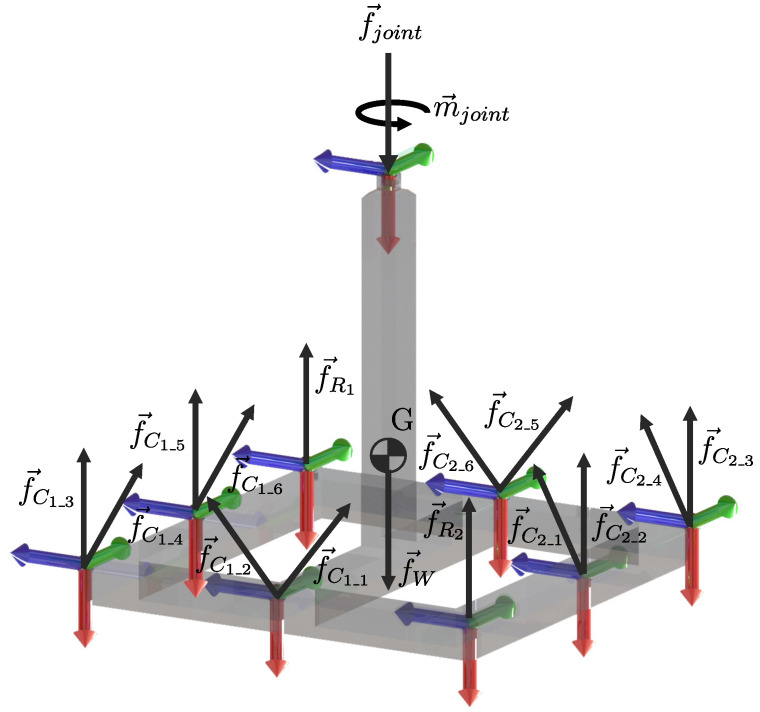
Free body diagram of the mobile platform.

Using the methodology proposed in [13], the fixed base and the flexible elements are replaced by the forces and moments exerted on the mobile platform, obtaining the free-body diagram shown in Figure 7.

Figure 7 shows all the moments and forces acting upon the mobile platform; the force labeled as ${\vec{f}}_{C_{1\_i}}$ and ${\vec{f}}_{C_{2\_i}}$ with i=1,2,…,6 represents the loads applied by the cables $C_{1}$ and $C_{2}$ over the mobile platform of the robot; the forces ${\vec{f}}_{R_{1}}$ and ${\vec{f}}_{R_{2}}$ express the loads applied by the tension springs $r_{1}$ and $r_{2}$, respectively; the force ${\vec{f}}_{W}$ represents the force exerted by gravity on the moving platform applied to its centroid, G. Likewise, the force-couple equivalent system composed of the force ${\vec{f}}_{joint}$ and the moment ${\vec{m}}_{joint}$ constitute the force and the moment of reaction that the universal joint exerts on the mobile platform.

The forces and moments exerted by the flexible elements on the mobile platform are defined as follows:

(48) $$\begin{array}{ccc} {\vec{f}}_{C_{1\_i}} & = & \left|{\vec{f}}_{C_{1}}\right|{\hat{C}}_{1\_i},\;\;\;\;\;\;\;\;{\vec{f}}_{C_{2\_i}}\;=\;\left|{\vec{f}}_{C_{2}}\right|{\hat{C}}_{2\_i}\\ {\vec{f}}_{R_{1}} & = & \left|{\vec{f}}_{R_{1}}\right|\hat{r_{1}},\;\;\;\;\;\;\;\;\;\;\;{\vec{f}}_{R_{2}}\;=\;\left|{\vec{f}}_{R_{2}}\right|\hat{r_{2}}\\ {\vec{f}}_{joint} & = & \left|{\vec{f}}_{joint}\right|{\hat{X}}_{1},\;\;\;\;{\vec{m}}_{joint}\;=\;\left|{\vec{m}}_{joint}\right|{\hat{X}}_{0}\\ {\vec{f}}_{W} & = & \left|{\vec{f}}_{W}=\right|{\hat{X}}_{0}\; \end{array}$$

where the unit vectors, ${\hat{C}}_{1\_i}$ and ${\hat{C}}_{2\_i}$ with i=1,2,3, …, 6, are obtained following the order shown in Figure 5 and from the direct kinematic position analysis provided by (32)–(43); the unit vectors $\hat{r_{1}}$ and $\hat{r_{2}}$ are calculated using the distribution of points shown in Figure 2; the unit vector ${\hat{X}}_{1}$ indicates the direction of the axis $X_{1}$ belonging to the reference frame $\Sigma_{1}$, observed from the reference frame $\Sigma_{0}$ obtained from the matrix TM0 defined by multiplying the matrices (2) and (3), while ${\hat{X}}_{0}={\left[\begin{array}{ccc} 1 & \;0 & \;0 \end{array}\right]}^{T}$.

Then, the equivalent force-couple system according to the forces and moments (48) is provided by [25]

(49) $$\begin{array}{ccc} \vec{f_{e}} & = & {\vec{f}}_{C_{1\_1}}+{\vec{f}}_{C_{1\_2}}+{\vec{f}}_{C_{1\_3}}+{\vec{f}}_{C_{1\_4}}+{\vec{f}}_{C_{1\_5}}+{\vec{f}}_{C_{1\_6}}+\ldots \\ & & {\vec{f}}_{C_{2\_1}}+{\vec{f}}_{C_{2\_2}}+{\vec{f}}_{C_{2\_3}}+{\vec{f}}_{C_{2\_4}}+{\vec{f}}_{C_{2\_5}}+{\vec{f}}_{C_{2\_6}}+\ldots \\ & & {\vec{f}}_{R_{1}}+{\vec{f}}_{R_{2}}+{\vec{f}}_{W}+{\vec{f}}_{joint} \end{array}$$

(50) $$\begin{array}{ccc} {\vec{m}}_{e_{O}} & = & {\vec{r}}_{S_{i\_1/0}}\times {\vec{f}}_{C_{1\_1}}+{\vec{r}}_{S_{i\_1/0}}\times {\vec{f}}_{C_{1\_2}}+{\vec{r}}_{M_{1/0}}\times {\vec{f}}_{C_{1\_3}}+\ldots \\ & & {\vec{r}}_{M_{1/0}}\times {\vec{f}}_{C_{1\_4}}+{\vec{r}}_{S_{i\_4/0}}\times {\vec{f}}_{C_{1\_5}}+{\vec{r}}_{S_{i\_4/0}}\times {\vec{f}}_{C_{1\_6}}+\ldots \\ & & {\vec{r}}_{S_{i\_3/0}}\times {\vec{f}}_{C_{2\_1}}+{\vec{r}}_{S_{i\_3/0}}\times {\vec{f}}_{C_{2\_2}}+{\vec{r}}_{M_{2/0}}\times {\vec{f}}_{C_{2\_3}}+\ldots \\ & & {\vec{r}}_{M_{2/0}}\times {\vec{f}}_{C_{2\_4}}+{\vec{r}}_{S_{i\_2/0}}\times {\vec{f}}_{C_{2\_5}}+{\vec{r}}_{S_{i\_2/0}}\times {\vec{f}}_{C_{2\_6}}+\ldots \\ & & {\vec{r}}_{R_{1/0}}\times {\vec{f}}_{R_{1}}+{\vec{r}}_{R_{2/0}}\times {\vec{f}}_{R_{2}}+{\vec{r}}_{G/0}\times {\vec{f}}_{W}+{\vec{m}}_{joint}\; \end{array}$$

where ${\vec{r}}_{S_{i\_1/0}}$, ${\vec{r}}_{M_{1/0}}$, ${\vec{r}}_{S_{i\_4/0}}$, ${\vec{r}}_{S_{i\_3/0}}$, ${\vec{r}}_{M_{2/0}}$, ${\vec{r}}_{S_{i\_2/0}}$, ${\vec{r}}_{R_{1/0}}$, ${\vec{r}}_{R_{2/0}}$, and ${\vec{r}}_{G/0}$ represent the distance vectors measured from the origin of the reference frame $\Sigma_{0}$ to the points $S_{i\_1}$, $M_{1}$, $S_{i\_4}$, $S_{i\_3}$, $M_{2}$, $S_{i\_2}$, $R_{1}$, $R_{2}$ and *G*, respectively.

The conditions that guarantee that the equivalent force-couple system described by (49) and (50) is in equilibrium are provided by

(51) $$\begin{array}{c} {\vec{f}}_{e}=\vec{0}\\ {\vec{m}}_{e_{O}}=\vec{0} \end{array}$$

By substituting the equivalent force-couple system provided by (49) and (50), under equilibrium conditions (51), a system of six equations with six unknowns is obtained. The solution of this system of equations provides the magnitudes of the forces and the moment, i.e., $|{\vec{f}}_{C_{1}}|$, $|{\vec{f}}_{C_{2}}|$, $|{\vec{f}}_{R_{1}}|$, $|{\vec{f}}_{R_{2}}|$, $|{\vec{f}}_{joint}|$ and $|{\vec{m}}_{joint}|$.

On the other hand, the magnitudes of the forces, ${\vec{f}}_{R_{1}}$ and ${\vec{f}}_{R_{2}}$, exerted by the springs on the mobile platform, can also be expressed as

(52) $$|{\vec{f}}_{R_{1}}|=k_{1}\cdot \delta_{1}$$

(53) $$|{\vec{f}}_{R_{2}}|=k_{2}\cdot \delta_{2}$$

where $\delta_{1}$ and $\delta_{2}$ are the longitudinal deformations of the springs $r_{1}$ and $r_{2}$, respectively, when they are subjected to an axial force. The longitudinal deformation of the springs is calculated by

(54) $$\begin{array}{ccc} \delta_{1} & = & L_{r_{1}}-L_{r_{1_{0}}}\\ \delta_{2} & = & L_{r_{2}}-L_{r_{2_{0}}} \end{array}$$

Using the obtained numerical values of (54) with the magnitudes of the spring forces $|{\vec{f}}_{R_{1}}|$ and $|{\vec{f}}_{R_{2}}|$, the stiffness constants of the springs $k_{1}$ and $k_{2}$ are calculated using (52) and (53), respectively, guaranteeing the static analysis of the proposed class 2 tensegrity robot.

### 2.4. Form-Finding Analysis

For the form-finding analysis of the proposed class 2 tensegrity robot, the methodology presented in [20] is applied, where the set of all possible geometric configurations of the robot provided by $\theta_{1}$ and $\theta_{2}$ are analyzed, and a subset of equilibrium configurations provided by $\theta_{e1}$ and $\theta_{e2}$ is determined.

The set of all possible configurations is established as follows: for each $\theta_{1_{i}}\in \left\{\theta_{1_{i}},\theta_{2_{i}}\right\}$ a set $\Lambda_{i}=\left\{\theta_{2_{j}}\right\}$ with i=1,2,…,n and j=1,2,…,n is defined by

(55) $$\begin{array}{c} \Lambda_{i}=\left\{\theta_{2_{1}},\theta_{2_{2}},\theta_{2_{3}},\ldots ,\theta_{2_{n}}\right\} \end{array}$$

Then, the subset of equilibrium configurations Ω is defined that must satisfy (51), and they are expressed by

(56) $$\Omega =\left\{\theta_{1_{i}},\Lambda_{i}:\;\vec{f_{e}}=\vec{0},\;{\vec{m}}_{e_{O}}≅\vec{0}\right\}$$

By solving the system of equations described in Equation (51), for each of the *n*-geometric configurations Ω, a set of forces and moments, Ψ, can be obtained, composed by

(57) $$\begin{array}{c} \Psi =\left\{\left|{\vec{f}}_{C_{1_{i}}}\right|,\left|{\vec{f}}_{C_{2_{i}}}\right|,\left|{\vec{f}}_{R_{1_{i}}}\right|,\left|{\vec{f}}_{R_{2_{i}}}\right|,\left|{\vec{f}}_{joint_{i}}\right|,\left|{\vec{m}}_{joint_{i}}\right|\right\}\; \end{array}$$

where Ψ is the set of forces and the moment corresponding to the *i*-geometric configuration analyzed. In addition to guaranteeing that the proposed class 2 tensegrity robot is in an equilibrium geometric configuration, it must also be ensured that it fulfills the conditions of a tensegrity robot according to [2]. With this purpose, the forces and the moment that constitute the set Ψ must also satisfy the following conditions:

(58) $$\begin{array}{ccc} \left|{\vec{f}}_{C_{1_{i}}}\right| & \geq & 0,\;\;\;\;\;\left|{\vec{f}}_{C_{2_{i}}}\right|\;\;\geq \;\;0\\ \left|{\vec{f}}_{R_{1_{i}}}\right| & \geq & 0,\;\;\;\;\;\left|{\vec{f}}_{R_{2_{i}}}\right|\;\;\geq \;\;0\\ \left|{\vec{f}}_{joint_{i}}\right| & \leq & 0,\;\;\left|{\vec{m}}_{joint_{i}}\right|\;\;≅\;\;0\; \end{array}$$

Then, considering (58), the set Ω in (56) is provided by

(59) $$\begin{array}{c} \Omega =\left\{\theta_{1_{i}},\Lambda_{i}:\left|{\vec{f}}_{C_{1_{i}}}\right|\geq 0,\left|{\vec{f}}_{C_{2_{i}}}\right|\geq 0,\left|{\vec{f}}_{R_{1_{i}}}\right|\geq 0,\right.\\ \left.\left|{\vec{f}}_{R_{2_{i}}}\right|\geq 0,\left|{\vec{f}}_{joint_{i}}\right|\leq 0,\left|{\vec{m}}_{joint_{i}}\right|≅0\right\}\; \end{array}$$

In this way, the set of joint coordinates Ω, represented in (59), contains all the equilibrium geometric configurations of the proposed class 2 tensegrity robot.

## 3. Numerical Example

As a study case, it is proposed to find the set Ω that contains the equilibrium configurations of the proposed class 2 tensegrity robot. The geometric specifications and initial conditions for the robot are shown in Table 2.

Substituting the data from Table 2 into (3)–(21), the coordinates of the points, M, $M_{1}$, $M_{2}$, $S_{i\_1}$, $S_{i\_2}$, $S_{i\_3}$, $S_{i\_4}$, $R_{1}$, and $R_{2}$ for the proposed class 2 tensegrity robot shown in Figure 3, are obtained.

**Figure 8:**
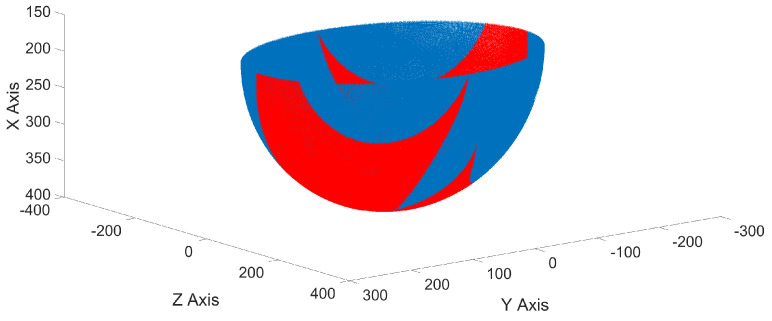
Three-dimensional workspace of the proposed class 2 tensegrity robot.

Evaluating (59) numerically with the data from Table 2 and the coordinates of the points shown in Figure 2, the matrix of points shown in Figure 8 is generated, where the blue points represent all possible geometric configurations of the proposed class 2 tensegrity robot, while the red dots represent all equilibrium geometric configurations that satisfy (51) and (58). Therefore, the red dots constitute the workspace of the proposed class 2 tensegrity robot.

**Figure 9:**
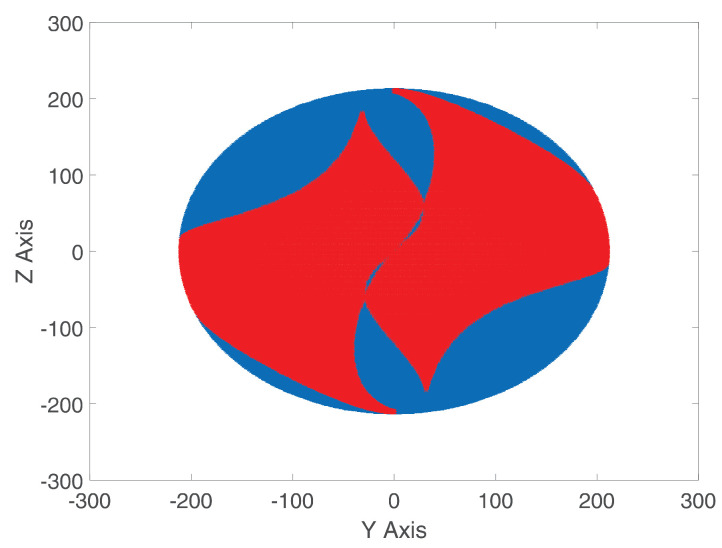
Workspace in the Y–Z plane of the proposed class 2 tensegrity robot.

Also, Figure 9 shows the geometric configurations analyzed in the Y–Z plane.

## 4. Numerical Experiments

This section compares the results of the numerical example with those obtained using software based on the finite element method, ANSYS^®^R18.2.

Consider four geometric configurations located within the workspace of the proposed class 2 tensegrity robot: equilibrium geometric configurations. Furthermore, they are arbitrarily selected on the workspace boundaries, whose values are shown in Table 3.

To generate the kinematic and mechanical behavior of the mobile platform of the proposed class 2 tensegrity robot, the following elements are used:

- The BEAM188 element is used to represent the rigid bars of the robot as they are suitable elements to analyze thin structures in three dimensions. The BEAM188 element has two nodes and six degrees of freedom at each node.
- The COMBIN14 element is used to represent both the wire segments $C_{i\_j}$, with i=1,2 and j=1,2,3,…,6, and the tension springs $r_{1}$ and $r_{2}$. The COMBIN14 element is suitable for modeling bodies subjected to uniaxial tension–compression loads. With proper constraints, the COMBIN14 element, having three degrees of freedom per node, is also used to represent combined spring-damper systems.

All types of elements used for discretizing the mobile platform and the flexible elements of the class 2 tensegrity robot in the simulation carried out in the commercial software ANSYS^®^R18.2 are shown in Table 4.

**Figure 10:**
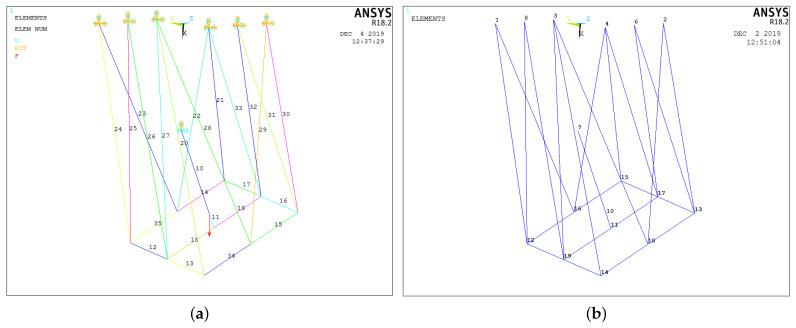
Class 2 tensegrity robot model in ANSYS^®^R18.2. (**a**) Element type. (**b**) Node numbering.

The information in Table 4 is shown graphically in Figure 10a. Figure 10b shows the nodes corresponding to the discretized elements. Nodes 1–8 represent the union of the flexible elements with the rigid base of the robot, and they are constrained both in translation and rotation in the three axes. Node 9 represents the universal joint whose boundary conditions restrict translation in the three axes as well as rotation about the *x*-axis. Node 10 represents the centroid of the mobile platform where the resultant of the external forces corresponding to gravity is applied. Nodes 11–19 are declared with no constraints or external forces applied to them.

**Figure 11:**
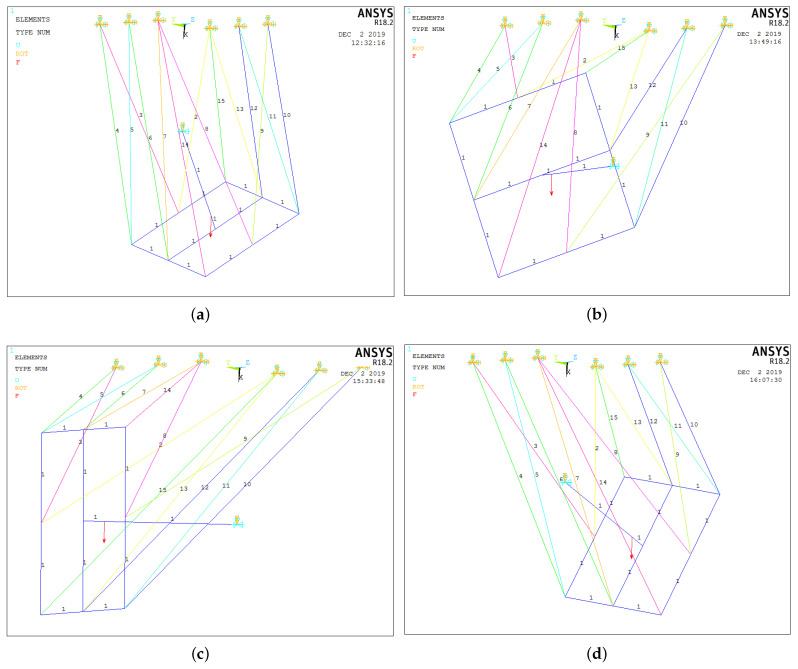
Geometric configurations analyzed. (**a**) Experiment 1. (**b**) Experiment 2. (**c**) Experiment 3. (**d**) Experiment 4.

Figure 11 shows the geometric configurations adopted by the platform of the robot corresponding to the joint coordinates listed in Table 3.

**Figure 12:**
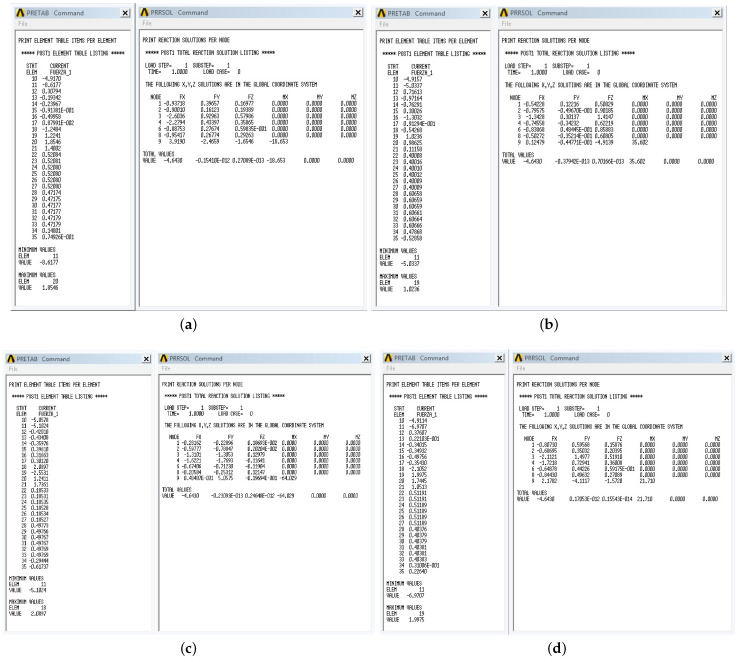
Static analysis results obtained from ANSYS^®^R18.2. (**a**) Experiment 1. (**b**) Experiment 2. (**c**) Experiment 3. (**d**) Experiment 4.

Screenshots of the static analysis results obtained using the software ANSYS^®^R18.2 are shown in Figure 12.

Moreover, Table 5 shows the comparison between the magnitudes of the forces, $\left|{\vec{f}}_{C_{1}}\right|$, $\left|{\vec{f}}_{C_{2}}\right|$, $\left|{\vec{f}}_{R_{1}}\right|$, $\left|{\vec{f}}_{R_{2}}\right|$, $\left|{\vec{f}}_{joint}\right|$, and $\left|{\vec{m}}_{joint}\right|$, obtained analytically, and the magnitudes of the same forces resulting from the simulation in the ANSYS^®^R18.2 software, corresponding to the four selected geometric configurations.

It has to be noted that the errors of the analytical and experimental results of ANSYS^®^ R18.2 are relatively small, so it is guaranteed that the equilibrium geometric configurations obtained satisfy the equilibrium conditions and the conditions that a tensegrity robot must fulfill.

## 5. Conclusions

In this paper, a hypothesis is presented where it is stated that, by increasing the number of interactions between the flexible elements formed by the cables with variable length, $C_{1}$ and $C_{2}$, and the rigid elements of the soft robot applied to a class 2 tensegrity robot, integrated by the mobile platform and the fixed base, as a consequence, there would be an increase in the number of equilibrium geometric configurations for the robot presented in [13]. For this purpose, a redesign of the configuration of the flexible elements in the class 2 tensegrity robot presented in [13] was proposed, remaining as shown in Figure 2. Subsequently, the proposed class 2 tensegrity robot was analyzed using the same methodology described in [13] to determine all the equilibrium geometric configurations of the robot, thus defining the workspace shown in Figure 8.

For the class 2 tensegrity robot presented in [13], it was determined that its workspace is composed of 215 equilibrium geometric configurations. Similarly, for the proposed class 2 tensegrity robot, it was found that the workspace, shown in Figure 8, comprises 50,223 equilibrium geometric configurations, representing an increase of 23,360% in the number of equilibrium geometric configurations. The significant increase in the workspace incurred by the class 2 tensegrity robot when integrating the modification in the interaction between the flexible and rigid elements allows for corroborating the hypothesis stated at the beginning of this research.

To verify the stability of the equilibrium geometric configurations found using the abovementioned technique, the software ANSYS^®^, based on finite element theory, was used to perform a series of numerical experiments. The results obtained showed that the analyzed geometric configurations constitute equilibrium geometric configurations that meet the definition of a tensegrity system [2]. Furthermore, the hypothesis of this manuscript was proved by numerical experiments; that is, the authors demonstrated that using the novel technique is an alternative to increasing the effective workspace in the class 2 tensegrity robot, which is different from the methods employed in [6,7,8,9,10,11,12], where their strategies were based on the concatenation of elementary tensegrity structures to increase the effective workspace and, consequently, the applications.

In future work, a mathematical model capable of describing the relationship between interaction points and geometric equilibrium configurations is planned. This model will consider variables such as point locations, inclination of flexible elements, and rigidity of said elements, among others. 

## Figures and Tables

**Table 2:** Form–finding parameters.

Definition	Variable	Value
Distance from the common point of the axes $\theta_{1}$ and $\theta_{2}$ to the moving platform	$a_{2}$	210 mm
Distance from the fixed base to the common point of the axes $\theta_{1}$ and $\theta_{2}$	$l_{1}$	190 mm
Width of the moving platform	$l_{2}$	220 mm
Initial condition of $\theta_{1}$	$\theta_{1,1}$	−1.5708 rad
Initial condition of $\theta_{2}$	$\theta_{2,1}$	−1.5708 rad
Joints coordinate increment	Δθ	0.0087 rad
Number of geometrical configurations analyzed	*n*	131,044
Initial *x* coordinate of the centroid, G, with respect to the reference frame $\Sigma_{W}$	$x_{ce}$	354.0442 mm
Initial *y* coordinate of the centroid, G, with respect to the reference frame $\Sigma_{W}$	$y_{ce}$	0 mm
Initial *z* coordinate of the centroid, G, with respect to the reference frame $\Sigma_{W}$	$z_{ce}$	76.4886 mm
Mass of the moving platform	*m*	0.4732 kg

**Table 3:** Arbitrarily selected positions.

Experiment Numbers	$\theta_{1}$ (rad)	$\theta_{2}$ (rad)
1	−0.5616	0.3432
2	−0.3441	1.5438
3	1.5612	0.0039
4	−1.0836	0.3258

**Table 4:** Types of elements.

Element Number	Physical Element	Element Type
1	Rigid elements	BEAM188
2–7	Cable $C_{1\_i}$	COMBIN14
8–13	Cable $C_{2\_i}$	COMBIN14
14	Spring $r_{1}$	COMBIN14
15	Spring $r_{2}$	COMBIN14

**Table 5:** Comparison between analytical and software results.

	Analytical	ANSYS^®^R18.2	Error
Experiment 1			
$\left|{\vec{f}}_{C_{1}}\right|$	0.52083 N	0.52080 N	0.0041%
$\left|{\vec{f}}_{C_{2}}\right|$	0.47178 N	0.47176 N	0.0024%
$\left|{\vec{f}}_{R_{1}}\right|$	1.85472 N	1.8546 N	0.0064%
$\left|{\vec{f}}_{R_{2}}\right|$	1.40022 N	1.4002 N	0.0014%
$\left|{\vec{f}}_{joint}\right|$	4.91739 N	4.9170 N	0.0079%
$\left|{\vec{m}}_{joint}\right|$	18.6549 N·mm	18.653 N·mm	0.0101%
Experiment 2			
$\left|{\vec{f}}_{C_{1}}\right|$	0.40010 N	0.40037 N	0.0691%
$\left|{\vec{f}}_{C_{2}}\right|$	0.60663 N	0.60661 N	0.0030%
$\left|{\vec{f}}_{R_{1}}\right|$	0.98630 N	0.98625 N	0.0050%
$\left|{\vec{f}}_{R_{2}}\right|$	0.11156 N	0.11158 N	0.01792%
$\left|{\vec{f}}_{joint}\right|$	4.91584 N	4.9157 N	0.0028%
$\left|{\vec{m}}_{joint}\right|$	35.61024 N·mm	35.602 N·mm	0.0231%
Experiment 3			
$\left|{\vec{f}}_{C_{1}}\right|$	0.18530 N	0.18529 N	0.0023%
$\left|{\vec{f}}_{C_{2}}\right|$	0.49770 N	0.49768 N	0.0030%
$\left|{\vec{f}}_{R_{1}}\right|$	1.24109 N	1.2411 N	0.0008%
$\left|{\vec{f}}_{R_{2}}\right|$	1.73608 N	1.7361 N	0.0011%
$\left|{\vec{f}}_{joint}\right|$	5.05786 N	5.0578 N	0.0011%
$\left|{\vec{m}}_{joint}\right|$	64.04513 N·mm	64.029 N·mm	0.0251%
Experiment 4			
$\left|{\vec{f}}_{C_{1}}\right|$	0.51191 N	0.51189 N	0.0023%
$\left|{\vec{f}}_{C_{2}}\right|$	0.40380 N	0.40379 N	0.0041%
$\left|{\vec{f}}_{R_{1}}\right|$	1.74458 N	1.7445 N	0.0045%
$\left|{\vec{f}}_{R_{2}}\right|$	1.05128 N	1.0513 N	0.0019%
$\left|{\vec{f}}_{joint}\right|$	4.91154 N	4.9114 N	0.0028%
$\left|{\vec{m}}_{joint}\right|$	21.71365 N·mm	21.710 N·mm	0.0168%

## Data Availability

Data are contained within the article.
